# Assessing Environmental DNA Detection in Controlled Lentic Systems

**DOI:** 10.1371/journal.pone.0103767

**Published:** 2014-07-31

**Authors:** Gregory R. Moyer, Edgardo Díaz-Ferguson, Jeffrey E. Hill, Colin Shea

**Affiliations:** 1 United States Fish and Wildlife Service, Conservation Genetics Laboratory, Warm Springs, Georgia, United States of America; 2 Program in Fisheries and Aquatic Sciences Tropical Aquaculture Laboratory, University of Florida, Ruskin, Florida, United States of America; 3 Department of Biology, Tennessee Technological University, Cookeville, Tennessee, United States of America; 4 Department of Fisheries and Allied Aquacultures, Auburn University, Auburn, Alabama, United States of America; Smithsonian's National Zoological Park, United States of America

## Abstract

Little consideration has been given to environmental DNA (eDNA) sampling strategies for rare species. The certainty of species detection relies on understanding false positive and false negative error rates. We used artificial ponds together with logistic regression models to assess the detection of African jewelfish eDNA at varying fish densities (0, 0.32, 1.75, and 5.25 fish/m^3^). Our objectives were to determine the most effective water stratum for eDNA detection, estimate true and false positive eDNA detection rates, and assess the number of water samples necessary to minimize the risk of false negatives. There were 28 eDNA detections in 324, 1-L, water samples collected from four experimental ponds. The best-approximating model indicated that the per-L-sample probability of eDNA detection was 4.86 times more likely for every 2.53 fish/m^3^ (1 SD) increase in fish density and 1.67 times less likely for every 1.02 C (1 SD) increase in water temperature. The best section of the water column to detect eDNA was the surface and to a lesser extent the bottom. Although no false positives were detected, the estimated likely number of false positives in samples from ponds that contained fish averaged 3.62. At high densities of African jewelfish, 3–5 L of water provided a >95% probability for the presence/absence of its eDNA. Conversely, at moderate and low densities, the number of water samples necessary to achieve a >95% probability of eDNA detection approximated 42–73 and >100 L, respectively. Potential biases associated with incomplete detection of eDNA could be alleviated via formal estimation of eDNA detection probabilities under an occupancy modeling framework; alternatively, the filtration of hundreds of liters of water may be required to achieve a high (e.g., 95%) level of certainty that African jewelfish eDNA will be detected at low densities (i.e., <0.32 fish/m^3^ or 1.75 g/m^3^).

## Introduction

Assessing the distribution, abundance, and dynamics of populations or species frequently requires the collection and identification of individuals from sample locations. As such, species detection is fundamental to scientific disciplines such as phylogenetics, conservation biology, and ecology. The idea of a species being either present or absent from a collection of sites has a long history in ecology, as it provides the foundation for assessing the status and dynamics of species at local and landscape scales. Reliable species detection during sampling, however, can be difficult to achieve, especially for species that are present in low abundances such as threatened and endangered taxa and, in some cases, newly invaded species [1]–[3].

Recent advances in molecular and forensic methods have provided innovative tools for detecting marine and aquatic organisms that may circumvent the aforementioned limitations [4]–[6]. One tool that holds particular promise is environmental DNA (eDNA). Defined as short DNA fragments that an organism leaves behind in non-living components of the ecosystem (i.e., water, air or sediments) [7]–[8], eDNA can be used to detect the presence (or absence) of a species through cells or tissues found in the environment containing the genetic material. In aquatic systems, genetic material can be collected via water filtration through a micron screen and tested for presence of the target species using specific genetic markers via polymerase chain reaction (PCR), quantitative PCR (qPCR) or direct sequencing of the PCR product. The basic technique outlined above raises the possibility to detect and monitor target taxa, particularly rare species, in aquatic environments while eliminating extraneous noise generated by the presence of (potentially numerous) non-target taxa. Consequently, eDNA has garnered increased attention for use with endangered aquatic organisms [2], [6] and aquatic invasive species [1], [9], [10].

Recently, there has been increased attention and scrutiny regarding eDNA detection methodologies [11]–[13]; yet, little consideration has been given to the utility and accuracy of eDNA presence/absence data with respect to rare or difficult-to-detect taxa [14], [15]. For example, what is the certainty of a species being detected via eDNA methods (i.e., what is the false positive error rate); in contrast, if a species fails to be detected using eDNA, then is it truly absent or is it present but simply not detected (i.e., what is the false negative error rate)? The latter, which is termed Process Type II Error [16], characterizes the imperfect detection of species and is of particular concern when using presence/absence data to make inferences regarding the predominant factors influencing the status, distribution, and dynamics of species. The confounded nature of non-detection and true absence imposes a fundamental problem when using eDNA presence/absence data, and failing to explicitly account for incomplete detection in a study design or analysis could lead to biased results and potentially unreliable inferences [17].

Occupancy modeling approaches are widely used in ecological research and management because they can effectively and efficiently account for potential biases associated with imperfect detection [18] and misidentification [19] of species. Occupancy models use records of species detections and non-detections during repeated surveys at a given location, and the resulting capture histories can be used to estimate model parameters of interest (e.g., detectability or occupancy). It follows that there is great potential for applying occupancy modeling approaches to eDNA detection/non-detection data [14], [17]. Incorporating such an approach into eDNA experimental designs could allow researchers to account for sources of bias such as false-negative [20] and false positive [19] measurement errors.

The African jewelfish (*Hemichromis letourneuxi*) is an aquatic invasive species that has spread throughout southern Florida since its introduction in 1965 [21]. More recently African jewelfish has spread into coastal river systems of west-central and southwest Florida and canals and wetlands along the Atlantic Coast from Cape Canaveral south [22]. Continued spread inland and northward in peninsular Florida is occurring (Hill, unpublished data). The species has the potential to compete with native [23], [24] and non-native species [25] and reduces survival of native fishes in seasonal refuges of short hydroperiod wetlands of the Florida Everglades [26], making its introduction and spread a threat to native ichthyofauna of Florida. In an effort to detect and monitor the spread of African jewelfish, eDNA markers have been established for the species [27]. Aquarium experiments using African jewelfish have shown a positive correlation between eDNA detection and fish density [27], with limited detection of eDNA (1 L water sample) occurring at densities less than 13 fish/m^3^. Difficulties associated with the detection of African jewelfish eDNA when densities are less than 13 fish/m^3^ is concerning given that the spread or introduction of this species could transpire at densities much lower than the observed value [28], [29]. Further, the issue of incomplete detection and the complexities of abiotic and biotic factors influencing eDNA detection raise more general questions regarding appropriate eDNA sampling strategies for monitoring programs focused on rare species and for understanding the spatial distribution of eDNA.

The goal of our study was to assess the detection of African jewelfish eDNA in a controlled lentic system at varying fish densities. Our specific objectives were to 1) determine the most effective water stratum for the detection of eDNA, 2) estimate true and false positive eDNA detection rates at varying fish densities, and 3) assess the number of water samples necessary to minimize the risk of false negative errors when developing eDNA sampling protocols.

## Materials and Methods

### Experimental Design

We used four artificial ponds as mesocosms to estimate and compare detection probabilities of the African jewelfish at differing densities. The four earthen ponds were located at the University of Florida's Tropical Aquaculture Laboratory, in Ruskin, Florida. The dimensions of each pond were approximately 18 m×7.5 m, with an average depth of 1.4 m. On June 10, 2013, 30 days prior to the introduction of fish, ponds were drained, pond bottoms and banks pressure washed, and remaining excess debris removed. Hydrated lime (CaOH) was then applied at a rate of 22.6 kg/pond to ensure that there were no remaining live fish in each pond. Over the course of 72 hrs, ponds were allowed to fill naturally from ground water. Once ponds were full, pH, dissolved oxygen, and temperature were monitored weekly. Three, 1-L eDNA water samples were taken from each pond 15 days before the introduction of the fish to check for any potential contamination. Samples were processed as outlined below, and African jewelfish DNA was absent in each pond of the study system before the fish introduction.

To begin the experiment, each of the four study ponds was stocked with a known number of fish (average TL = 69.92 mm; average wt. = 5.36 g): pond I contained 0 fish (control), pond II contained 60 fish (low density: 0.32 fish/m^3^ or 1.7 g/m^3^), pond III contained 330 fish (moderate density: 1.75 fish/m^3^ or 9.35 g/m^3^), and pond IV contained 990 fish (high density: 5.24 fish/m^3^ or 28.08 g/m^3^; Table 1). Note that all animal research was approved by the University of Florida, Institute of Food and Agriculture Sciences, Animal Research Committee (Approval # 002-13RUS). We stratified each pond into nine transects, three of which were located in one third of the pond (section 1), three in the middle third (section 2), and three in the remaining third (section 3). Three sample locations were then selected along each transect and randomly assigned one of three water column positions to each sample: surface, middle, or bottom. Following the first 24 hrs (i.e., day 1), 27 1-L water samples were collected from each pond at the specified locations using a Van Dorm collection bottle to ensure adequate coverage (depth and surface area). A kayak was used to move between transects and quadrants and was cleaned between ponds with Alconox detergent (1∶100 dilution, Alconox, Inc) to avoid contamination. This protocol was repeated on days 5 and 10, for a total of 81 samples in each pond and 324 samples total. On each sampling day, pond temperature was recorded.

**Table 1:** Pond number, pond volume, fish density, number of fish stocked, average temperature, and number of African jewelfish detections by water stratum across 27 water samples conducted on days 1, 5, and 10 of the study.

Pond	Day	Volume (m^3^)	Density (no. m^−3^)	Number fish	Temperature (C)	Detections by strata		
						Surface	Middle	Bottom
1	1	189	0	0	30.20	0	0	0
1	5	189	0	0	30.17	0	0	0
1	10	189	0	0	28.3	0	0	0
2	1	189	0.32	60	30.77	0	0	0
2	5	189	0.32	60	29.77	0	0	0
2	10	189	0.32	60	28.23	0	1	0
3	1	189	1.75	330	30.7	1	1	0
3	5	189	1.75	330	29.87	0	0	0
3	10	189	1.75	330	28.23	0	0	0
4	1	189	5.24	990	30.77	2	2	3
4	5	189	5.24	990	29.77	2	0	1
4	10	189	5.24	990	28.3	6	2	7

### Molecular methods

Each water sample was treated with 1 mL of 3M sodium acetate (pH 5.2) and 33 mL 95% non-denature ethanol for DNA/tissue preservation and refrigerated on site until filtration. Each water sample was filtered on site and filter paper frozen until extraction date. DNA was extracted following the protocol of Díaz-Ferguson et al. [27], however; the MOBIO Power Water DNA Isolation kit was substituted for the Rapid Water Isolation kit. Final DNA templates were eluted in 45 uL of buffer provided with the kit and then an ethanol precipitation was conducted to improve quality and concentration of the yielded DNA.

Taqman qPCR assays were employed to detect the presence of African jewelfish eDNA in each water sample collected from the four experimental ponds using primers AJFq3 and AJFRq2 and probe Pr028373859 designed for the target species (Díaz-Ferguson et al. 2014). Taqman assays were optimized for 20 uL reactions using DNA normalized to a concentration of 25 ng/uL and Taqman core reagents as follows: 2.0 uL of 5× *Taq* reaction buffer (Applied Biosystems, Inc), 2.5 uL MgCl_2_ (25 mM), 0.5 uL of each dNTP (1 mM), 1 uL of each primer (10 uM each), 0.20 uL probe Pr028373859 (10 uM), 0.5 uL AmpErase (Uracil-N-glycosylase), and 0.20 uL Amplitaq Gold *Taq* polymerase (5 U/uL, Applied Biosystems, Inc).

All assays were conducted using the following thermal profile: 60 C (1 min), initial denaturation at 95°C for 10 min., followed by 35 cycles of 95 C (15 s) and 60 C (1 min.) Detection of DNA from each sample was performed using a 7500 Fast Real Time PCR machine (Applied Biosystems, Inc.). Taqman assay quality controls consisted of repetition of all qPCR results and inclusion of two negative qPCR controls (substitution of distilled water for DNA) and a positive qPCR inhibition control for each qPCR plate. The positive control consisted of a water sample taken from each pond, spiked with 5–10 mg/uL lyophilized tissue from *H. letourneuxi*, filtered, DNA extracted, and DNA used as a positive confirmation that our qPCR reactions were working correctly in the presence of potential inhibitors. In addition, we sequenced 25% of the positive Taqman assays (following the protocol outlined by Díaz-Ferguson et al. 2014) for confirmation that the qPCR product was truly that of African jewelfish. All sequences were imported into GENEIOUS v4.8.5 alignment editor (Biomatters, available from http://www.geneious.com/), ends trimmed, aligned by eye, and compared for base pair composition and similarity with other African jewelfish sequences previously deposited in the GenBank.

### Statistical analyses

Our primary interest was to assess per-sample eDNA detection rates (i.e., prevalence of false-negative errors); however, we also evaluated the prevalence of false positive errors in the sample data. False-negative errors represented instances where, for a given 1 L water sample, qPCR DNA amplification failed to detect African jewelfish when it was known to be present in a pond. In contrast, false-positive errors represented instances where the species was detected when it was known to be absent from a pond (i.e., the control pond). To estimate eDNA detection probabilities, we fitted logistic regression models [30] relating eDNA detection/non-detection data to two pond-level factors, fish density and water temperature, and one sample-level factor, position in the water column. Water temperatures varied among ponds and time periods; however, there was a general decline in temperature across all ponds over the 10-day study period owing to a cold front that moved through the region. We assumed that fish density remained constant over the course of the study. We observed no fish mortality in any pond over the 10-day period; hence, we believe that the assumption of constant fish density was valid. For both data types, the dependent variable was the detection or non-detection (binary coded) of eDNA from individual water samples. Note that we excluded control pond data from the analysis of eDNA detection because the pond did not contain fish. Conversely, only the control pond data were used to estimate false positive error rates, because any positive eDNA detections in the control pond were, by definition, false positives.

Regular logistic regression cannot account for dependence (i.e., autocorrelation) among repeated samples, and we suspected that repeated water samples taken from particular sections and transects were dependent [31]. For example, it was possible that individuals, and hence their eDNA, were concentrated in particular areas of each pond, which would tend to inflate false negative errors as eDNA would not be present in some areas and, hence, unavailable for detection. To account for dependence among samples, we fitted hierarchical logistic regression models to the DNA detection/non-detection data [32]. For our study, the log-odds of eDNA detection, 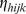, was modeled as:
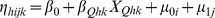
where 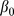 was the intercept, 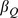 was the effect of pond- (*h*) or sample-level (*k*) factors (fish density, water temperature, and water column position) 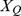 on eDNA detection, and 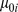 and 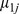 were the section- (*i*) and transect (*j*)-level random effects, respectively, that were assumed to be normally distributed with mean of zero and random effect-specific variance [33]. The random components *u_0i_* and *u_1j_* represented unique effects associated with sections and transects, respectively, that were unexplained by pond- and sample-level covariates. Because there were no DNA detections in the control pond (see Results), it was not possible to model false positive errors as a function of covariates. Thus, we fit a single “intercept only” logistic regression model (i.e., the log-odds of false positive errors, 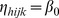) to estimate the false positive error rates and estimated the likely number of false positive errors in the false negative data (i.e., data from ponds with fish) assuming a sample size of 243. We used Markov Chain Monte Carlo (MCMC) as implemented in OpenBUGS software, version 3.2.1 [34] to fit candidate hierarchical logistic regression models. All models were fit using 200,000 iterations, a 50,000 iteration burn in (i.e., the first 50,000 MCMC iterations were dropped), and diffuse priors.

We used an information-theoretic approach [35] to evaluate the relative fit of candidate models relating pond- and sample-level characteristics to eDNA detection/non-detection data. For data from the three ponds that were stocked with fish, we developed 16 models representing relations between various combinations of pond- and sample-specific predictors and eDNA detection. The pond temperature predictor represented the measured temperature at the surface, middle, and bottom of each pond during each sample day, which resulted in three temperature measurements per pond on days 1, 5, and 10 of the study; the fish density predictor represented the known density of fish in each pond (low, moderate, high); and the water column position predictors included ‘middle’ and ‘bottom’, with ‘surface’ samples serving as the statistical baseline. The categorical water column position predictors were binary coded as 0 (surface) or 1 (middle and bottom). To facilitate model-fitting, we standardized both continuous predictors, water temperature and fish density, with mean of zero and standard deviation of one.

Prior to fitting candidate models, we evaluated the relative-fit of four different variance structures using the global (all predictors) model by fitting models that contained several combinations of random effects for sections and transects. The four variance structures included (1) no random effects, (2) a random intercept associated with individual sections, (3) a random intercept associated with individual transects, and (4) a random intercept associated with individual sections and transects. The best approximating variance structure was identified using the Deviance Information Criterion (DIC). The DIC is a Bayesian measure of model fit or adequacy, with smaller DIC indicating a better approximating model [36]. We then evaluated the relative fit of the 16 candidate models using DIC and calculated DIC weights following Link and Barker [37], which range from 0–1 with the best approximating candidate model having the highest weight. We considered the most plausible models to be those with DIC weights within 10% of the best-approximating model, which is similar to Royall's general rule-of-thumb of 1/8 or 12% for evaluating strength of evidence [38].

We assessed the precision of parameter estimates for each model by calculating 95% Bayesian credible intervals [39], which are analogous to 95% confidence intervals. To facilitate interpretation, we also calculated odds ratios (OR) for each fixed-effect pond- and sample-level predictor variable [40]. We assessed MCMC convergence for each model in the confidence set using the diagnostics detailed by Gelman and Rubin [41]. Lastly, we assessed the adequacy of the global model (Goodness of fit) by calculating a Bayesian p-value using the discrepancy measure method [42]. Extreme Bayesian p-values (i.e., ≤0.05 or ≥0.95) indicate that a model does not adequately describe the data.

Using parameter estimates from the best-approximating model, we also calculated cumulative detection probabilities to evaluate the number of 1-L water samples required to achieve a specified level of certainty that low, moderate, and high density African jewelfish populations would be detected at least once. We also calculated per-sample detection probabilities as a function of fish density.

## Results

A total of 28 detections of African jewelfish eDNA were made across 324 individual 1-L water samples collected from the four experimental ponds (Table 1). Sequence confirmation of qPCR fragments showed between 83–89% query coverage (percent of the query sequence that overlaps the subject sequence) and a percent sequence similarity between 92–96% that corresponded to *Hemichromis* (GenBank accession numbers: KJ553580.1, JQ667546.1, JN026744, GU817297.1, AY662793.1, KJ553529.1).

Excluding 81 water samples collected from the control pond (i.e., no fish present), there were 28 detections across the 243 collections taken from ponds that contained varying densities of fish, which corresponded to an overall eDNA detection rate of ∼12%. The detection of eDNA was more prevalent on the surface and bottom (each with 39% of the detections) when compared to the middle of the water column. Notably, there were no false positive detections among the 81 control samples.

The best-approximating error structure for the logistic regression models relating eDNA detection/non-detection data to pond- and sample-level covariates included no random effects associated model intercepts and slopes, indicating no substantial dependence among pond transects or quadrants. The assessment of model adequacy using the discrepancy measure method indicated that the global model provided an adequate description of the data, with a Bayesian p-value of 0.58. The confidence model set consisted of four models that contained various combinations fish density, water temperature, and sample position in the water column (Table 2). The best-approximating model contained density, temperature, and middle and was 1.44, 2.44, 3.54, and 5.57 times more plausible than the next best-approximating models in the confidence set (Table 2).

**Table 2:** Deviance, effective number of parameters (pd), deviance information criterion (DIC), ΔDIC, DIC weights (w*_i_*), and Bayesian p-values (p-value) for the confidence set of logistic regression models relating African jewelfish eDNA detections to pond- and sample-level factors.

Model	Deviance	pd	DIC	ΔDIC	w*_i_*	p-value
Intercept, middle, density, temperature	125.70	3.69	129.39	0.00	0.39	0.60
Intercept, density, temperature	127.20	2.95	130.15	0.75	0.27	0.60
Intercept, middle, bottom, density, temperature	126.50	4.63	131.13	1.74	0.16	0.58
Intercept, bottom, density, temperature	127.90	3.94	131.84	2.45	0.11	0.58
Intercept, middle, density	130.00	2.71	132.71	3.32	0.07	0.57

Parameter estimates from best-approximating model indicated that the per-sample probability of eDNA detection was strongly and positively related to fish density and negatively related to water temperature (Table 3). Odds ratios (OR) indicated that African jewelfish were 4.86 time more likely to be detected for every 1 SD (2.53 fish/m^3^) increase in fish density, whereas the species was 1.67 times less likely to be detected for every 1 SD (1.02 C) increase in pond temperature (Table 3). Parameter estimates for the remaining covariates in the confidence model set, bottom and middle, indicated that per-sample detection was highest in collections taken from the surface (i.e., detection was negatively related to middle and bottom); however, the parameter estimates were considered imprecise as the 95% credible intervals contained zero (Table 3).

**Table 3:** Parameter estimates (Mean), standard deviations (SD), 95% credible intervals, and odds ratios (OR) from the confidence set of logistic regression models relating African jewelfish eDNA detections to pond- and sample-level factors.

Model	Parameter	Mean	SD	Lower 95%	Upper 95%	OR
Best						
	Intercept	−2.78	0.39	−3.62	−2.08	
	Middle	−0.81	0.50	−1.83	0.14	0.44
	Density	1.58	0.30	1.04	2.22	4.86
	Temperature	−0.51	0.23	−0.97	−0.07	0.60
2nd best						
	Intercept	−2.98	0.38	−3.78	−2.31	0.05
	Density	1.56	0.30	1.02	2.19	4.75
	Temperature	−0.51	0.23	−0.96	−0.08	0.60
3rd best						
	Intercept	−2.70	0.45	−3.64	−1.88	
	Middle	−0.91	0.55	−2.01	0.15	0.40
	Bottom	−0.23	0.50	−1.21	0.74	0.80
	Density	1.60	0.30	1.05	2.23	4.93
	Temperature	−0.53	0.23	−1.00	−0.08	0.59
False positive						
	Intercept	−4.20	0.78	−5.91	−2.86	

Also reported is the single parameter estimate (intercept) associated with the logistic regression model fit to the control data to estimate the probability of false positive errors.

Although there were no false positive errors associated with the control data in this study, it was still possible to estimate the probability and number of false positive errors under the assumption of binomially distributed data with a sample size of 81 (i.e., the number of 1-L water samples in the control pond). The parameter estimate from the logistic regression model fit to the control data indicated that false positive errors were very unlikely, with an estimated per-sample probability of 0.014 (Table 4). Across the 243 1-L water samples conducted in the three stocked ponds, the per-sample false-positive error rate estimate of 0.014 suggested that the likely number of false positives averaged 3.62 and ranged from 0–12.

**Table 4:** Parameter estimates (Mean), standard deviations (SD), and 95% credible intervals for the logistic regression model relating African jewelfish eDNA detections to pond- and sample-level factors.

Parameter	Mean	SD	Lower 95%	Upper 95%
Intercept	−3.25	0.52	−4.33	−2.29
Middle	−0.86	0.50	−1.89	0.08
Density	1.54	0.29	1.01	2.17
Day	0.10	0.06	−0.01	0.22
Deviance	126.70	2.853		
DIC	130.76			

This model is identical to the best approximating model listed in Table 3 but with time (day) substituted for temperature.

**Figure 1:**
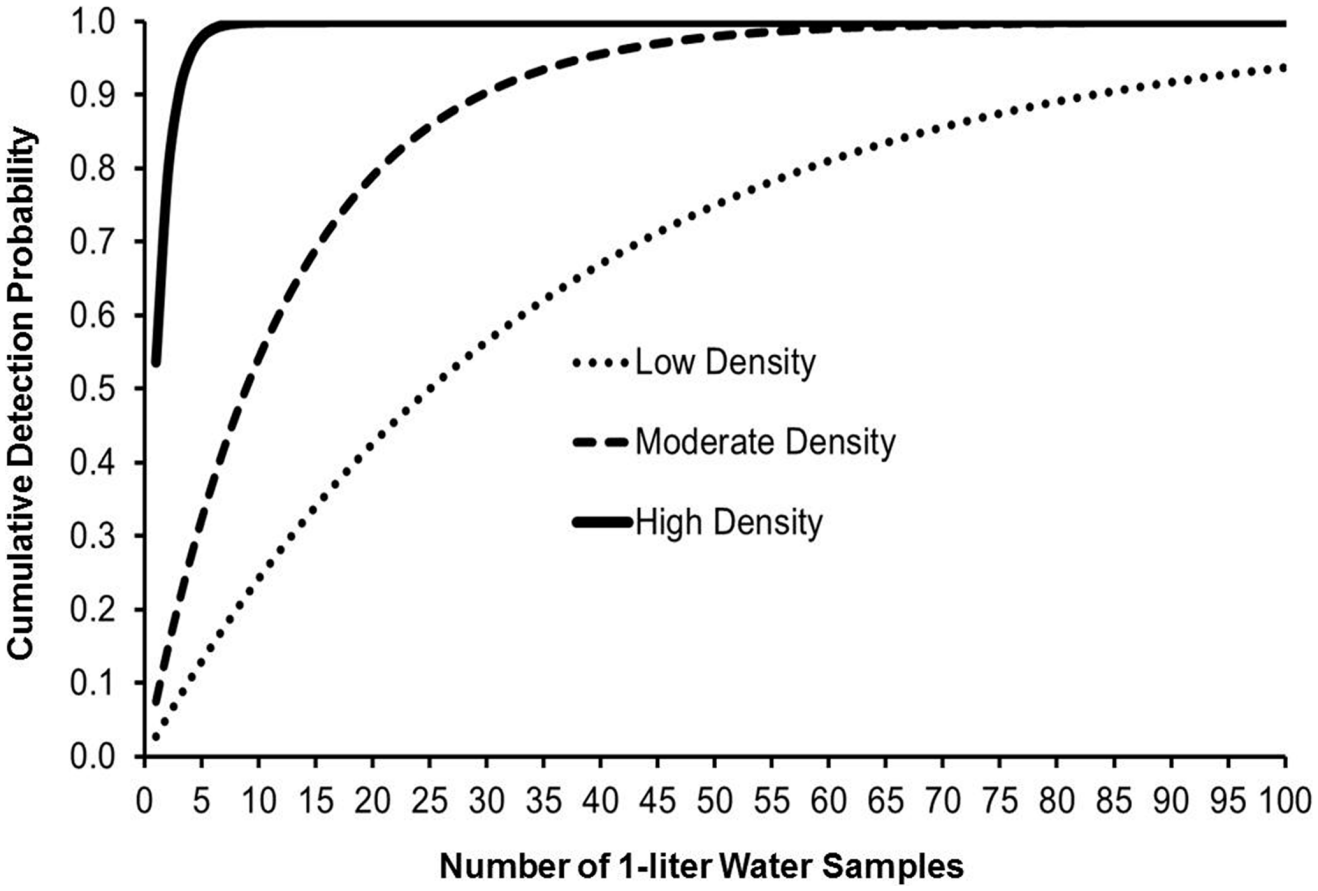
Predicted cumulative African jewelfish eDNA detection probability with an increasing number of 1-L water samples. Detection estimates are based on parameter estimates from the best-approximating hierarchical logistic regression models relating African jewelfish eDNA detection/non-detection data to pond- and sample-level covariates and were calculated for low density (0.32 fish/m^3^), moderate density (1.75 fish/m^3^), and high density (5.24 fish/m^3^) populations assuming a water temperature of 28 C.

**Figure 2:**
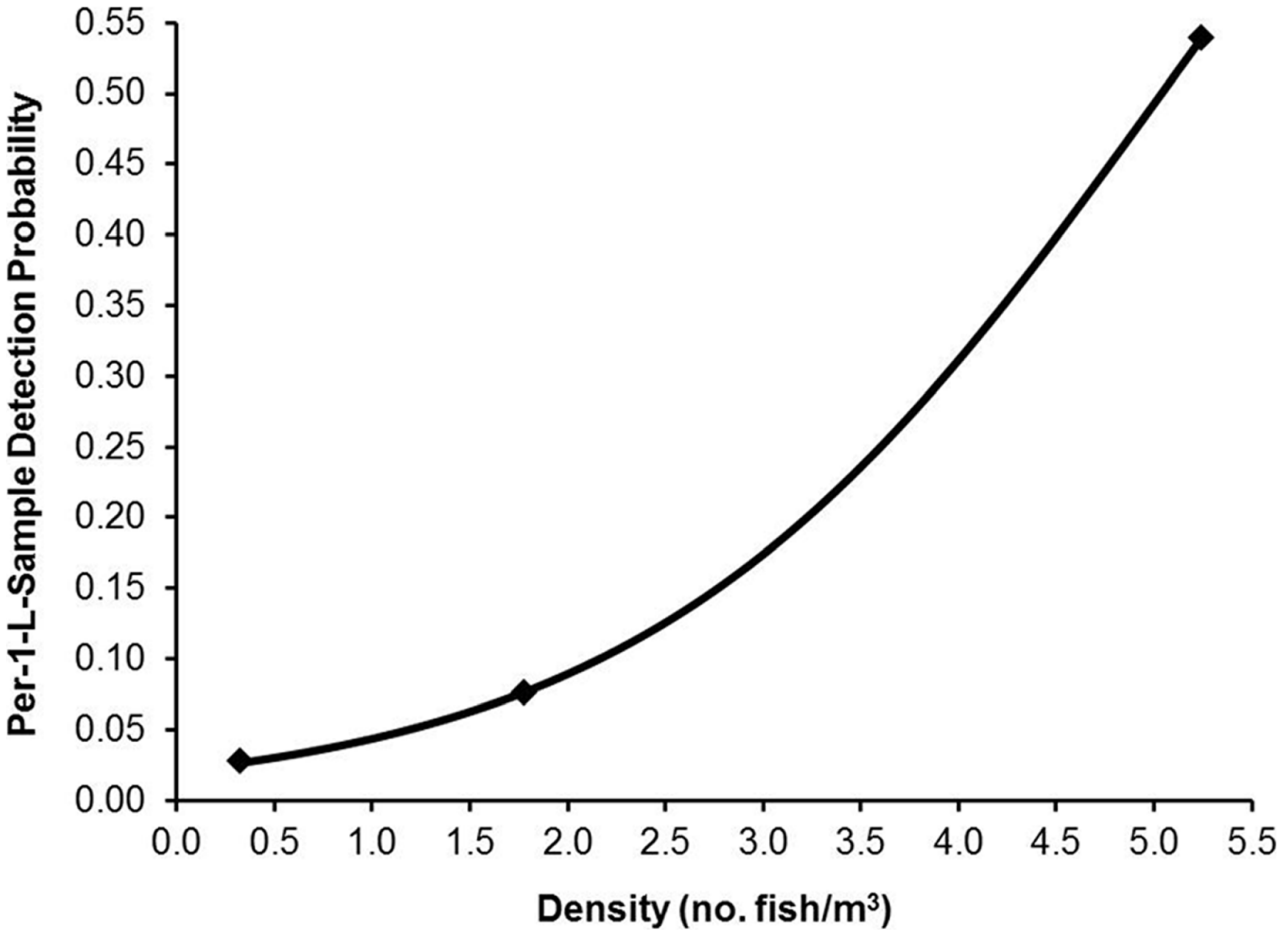
Predicted per-1-L water sample DNA detection probability with increasing densities of African jewelfish in experimental ponds. Detection estimates are based on parameter estimates from the best-approximating hierarchical logistic regression models relating African jewelfish eDNA detection/non-detection data to pond- and sample-level covariates. Filled diamonds represent the low (0.32 fish/m^3^), moderate (1.75 fish/m^3^), and high densities (5.24 fish/m^3^) used in this study.

Using parameter estimates from the best-approximating model, the number of 1-L water samples required to achieve a specified level of certainty varied considerably depending on the density of African jewelfish aggregates (Figure 1). Per-sample detection probabilities plotted as a function of fish density demonstrated that although the per-sample probability of detection increased with fish density, the relationship was nonlinear across the range of densities used in this study (Figure 2).

Note that our best-approximating model included temperature and not time; yet, we expected a positive relationship between time and eDNA detection. Unfortunately it was difficult to differentiate the effects of temperature and time because temperature declined over the 10-day period in the entire system (Table 1). To address this, we constructed a model that included time instead of temperature. We observed a weak positive relationship (i.e., for every 1 day increase, the log-odds of detection increased by 0.10; Table 4) and based on the DIC (130.76 vs. 129.39), the time model had less support than the temperature model in explaining eDNA detection. Given our findings and that the time since invasion is rarely known, we opted to use temperature in the models because it was more relevant to our study objective.

## Discussion

The ability to detect individuals at low densities in aquatic habitats is critical for successful control and management of invasive species [43] and for the conservation of threatened and endangered organisms [3], [44]. Unfortunately, rarity typically presents problems when dealing with both spatial sampling and detectability [44]. This issue is not new and like traditional sampling methods designed to detect rare or elusive species, eDNA sampling methods will suffer the same biases and problems. Therefore the development of methods and models that properly account for imperfect detection of eDNA should be a vital first step in designing and implementing detection and monitoring surveys for rare organisms that rely on eDNA methods [16], [17], [45].

While our study and numerous others have illustrated a positive and often significant relationship between organismal density and eDNA detection [14], [17], [27], [46], our basic understanding of the biotic and abiotic factors influencing eDNA detection is still in its infancy [however, see 47], with the majority of studies focusing on type I and II errors associated with the molecular method itself [e.g.], [3], [11,16]. In contrast, the focus of our study was to assess the false negative error rate termed Process Type II Error by Darling and Mahon [16]. While Darling and Mahon [16] recognized that the estimation of false negative and false positive error rates is important for eDNA assay development, they acknowledged that few if any studies effectively address this issue. Our study sought to provide a quantitative approach for estimating and understanding sampling efficiency for African jewelfish.

The eDNA from living macrofauna most likely originates from urine and feces, epidermal tissues, or other secretions such as reproductive fluids and reproductive cells. Most of this material is introduced into the water column as large particles (>1000 um) that remain at the surface for a limited amount of time before sinking or breaking apart [13], [48]; thus, the surface provides a logical place to survey for eDNA and it is also relatively efficient to collect surface samples when compared to soil samples from the bottom of a lentic system. While eDNA studies have concentrated sampling efforts near the surface [3], [49], none have justified their sampling approach. Once introduced, African jewelfish seek and remain on the bottom of an earthen pond (J. Hill personal observation); therefore, we suspected that samples taken from the bottom would show a significant increase in eDNA detection. In contrast, our results indicated that the best section of the water column to sample and detect eDNA was the surface and to a lesser extent the bottom. Our findings support the pattern that, at least in small lentic systems, eDNA remains at the surface level for a given time period before settling to the bottom or until degradation occurs.

Elevated temperature can accelerate the rate of eDNA degradation. Degradation can occur directly by denaturing the DNA or indirectly by increasing enzymatic activity and microbial metabolism [47]. Parameter estimates from our best-approximating model indicated that eDNA detection was negatively related to water temperature such that the species was 1.67 times less likely to be detected for every 1 SD (1.02 C) increase in stream temperature. A similar observation was found for preliminary eDNA persistence trials of African jewelfish held in aquaria. In these trials, African jewelfish eDNA was found to degrade between 25 and 33 C (E. Díaz-Ferguson unpublished data). While the negative effect of temperature in our study likely reflects the combined effects of lower degradation rates under lower temperatures and more eDNA in the system as time progressed, the influence of time on eDNA detection was assumed minimal – a finding supported by Díaz-Ferguson et al. [27] who found a non-significant relationship between African jewelfish eDNA detection and time in aquaria held at a constant temperature over a seven day period. Thus, we believe that temperature was a significant factor influencing eDNA detection in our study. This finding suggests that to minimize the negative influence of temperature on species detection rates, the implementation of eDNA monitoring programs in the relatively warm waters of the tropics and subtropics should be cautioned if ambient water temperatures exceed 29–30 C.

At high densities of African jewelfish (5.24 fish/m^3^), the filtration of 3–5 L of water (or the filtration of 3–5, 1-L water samples) should provide a high degree of confidence (95–100% probability) to confirm the presence or absence of its eDNA. However, if only a 1-L water sample was collected from our pond containing 990 fish, then our ability to detect eDNA would be approximately 55%. For our ponds that contained 330 and 60 fish, we had a 7% and a 3% chance of detecting African jewelfish eDNA if a 1-L water sample was taken. Conversely, at moderate and low densities, the number of water samples necessary to achieve a 95–100% probability of eDNA detection would approximate 42–73 L and >100 L, respectively. Our findings highlight a well-known and important concept with hypothesis testing – statistical power [31]. Scientist and resource managers using eDNA methods must agree on the level of accepted error prior to hypothesis testing [16], but depending on the hypothesis being tested, they will often want to keep both type I and type II errors small. Clearly, then, the only way to minimize eDNA false negatives is to improve the power of the test while keeping the significance level (α) constant. To do so requires increasing the sensitivity ( = power) of the test either by increasing the sample size (volume of water tested) or by increasing the sensitivity of the eDNA marker. Our eDNA marker appears sufficiently sensitive because the observed theoretical lower limit of qPCR detection using our eDNA marker was similar to other eDNA studies [27]; therefore, if minimizing type I and type II errors is a priority when using eDNA to monitor the leading edge of invasion for African jewelfish, then a large volume of water must be screened.

Our findings raise a vexing problem when designing eDNA sampling strategies for aquatic species that are rare – the density of the organism is usually unknown; hence, the amount of water necessary to detect the organism with a high level of certainty also will be unknown. Minimizing false negatives (i.e., increasing detection probability) will come at the cost of filtering more water (either in the form of more volume or more samples). The filtration of hundreds of liters of water through a small micron filter tends to be problematic (filters clog) and often expensive (e.g., filtration of 100, 1-L bottles each through its own filter); alternatively, devices that maximize water volume such as inline filters or plankton nets, may prove valuable for eDNA monitoring of rare species [13], but will be contingent on the particle size of aqueous eDNA being emitted from the organism of concern [13].

While our study was not specifically designed to address the issue of taking numerous smaller samples vs. one large sample (e.g., 50, 1-L water samples vs 1, 50-L water sample), we recommend the former, especially for rare species in lentic systems. In a lentic environment, the taxon in question may have specific (patchy) habitat requirements; thus, stratifying by habitat and conducting numerous smaller-volume samples would be preferred over taking one large-volume sample. Furthermore, more habitat and sample-specific covariates (i.e., all the interesting heterogeneity within the sampling site) are available for potential use in an occupancy model when collecting numerous smaller-volume samples vs. obtaining one large-volume sample.

Finally, our study emphasizes the difficulties of inferring detection probabilities for an organism inhabiting a natural system simply from aquarium trials. First, even when one fish is placed in an aquarium, it is difficult to simulate lower densities necessary for inferring accurate detection probabilities (i.e., there is a nonlinear relationship between eDNA detection and density, see Figure 2). For example, Díaz-Ferguson et al. [27] used one fish (5.45 g) in a 75.5 L aquarium to simulate their lowest density; however, this approximated to a density of 13 fish/m^3^ (or 70.85 g/m^3^) in our pond experiment and was a value greater than our highest density pond. Thus, if the species of concern is rare, then the estimation of detection probabilities should be conducted in a larger controlled system that can simulate the rarity of the organism in its natural setting. For an organism inhabiting a lotic system, the use of artificial streams [50] or raceways may be necessary. Alternatively, the estimation of detection probabilities may be unattainable for an endangered organism due to its rarity; thus, the use of a surrogate species (e.g., a close congener with similar life history attributes) may be necessary. Second, aquarium trial experiments of African jewelfish failed to detect eDNA from a 1-L water sample at a density of 13 fish/m^3^ [27]. In the present study, a 1-L water sample taken at this density should always detect African jewelfish; thus, aquarium experiments appear to have underestimated the detection of African jewelfish eDNA. There are a variety of potential explanations for the discrepancy in detection probabilities including behavioral (antagonistic behavior, increased movement) and environmental (wind/wave action); regardless, our study demonstrated the complexities of extrapolating eDNA detection probabilities from a controlled to a natural environment.

Our results and those of Díaz-Ferguson et al. [27] indicate that detection probabilities for African jewelfish can be imperfect (i.e., <1) and vary spatially or temporally in response to local environmental conditions. As such, presence-absence data derived from eDNA-based methods (e.g., the proportion of sites where a species was detected) where the density of African jewelfish is low will be negatively biased and could have profound implications when determining the leading edge of invasion for this species if imperfect detection is not taken into account. Potential biases associated with incomplete detection could be alleviated by formally estimating detection probabilities under an occupancy modeling framework [51], [52]; alternatively, the filtration of hundreds of liters of water may be required to detect African jewelfish at low densities (i.e., <0.32 fish/m^3^ or 1.7 g/m^3^) with a desired level of confidence.
